# The clinical spectrum of the congenital myasthenic syndrome resulting from *COL13A1* mutations

**DOI:** 10.1093/brain/awz107

**Published:** 2019-05-13

**Authors:** Pedro M. Rodríguez Cruz, Judith Cossins, Eduardo de Paula Estephan, Francina Munell, Kathryn Selby, Michio Hirano, Reza Maroofin, Mohammad Yahya Vahidi Mehrjardi, Gabriel Chow, Aisling Carr, Adnan Manzur, Stephanie Robb, Pinki Munot, Wei Wei Liu, Siddharth Banka, Harry Fraser, Christian De Goede, Edmar Zanoteli, Umbertina Conti Reed, Abigail Sage, Margarida Gratacos, Alfons Macaya, Marina Dusl, Jan Senderek, Ana Töpf, Monika Hofer, Ravi Knight, Sithara Ramdas, Sandeep Jayawant, Hans Lochmüller, Jacqueline Palace, David Beeson

**Affiliations:** 1 Neurosciences Group, Nuffield Department of Clinical Neurosciences, Weatherall Institute of Molecular Medicine, University of Oxford, Oxford, UK; 2 Nuffield Department of Clinical Neurosciences, John Radcliffe Hospital, University of Oxford, Oxford, UK; 3 Departamento de Neurologia, Faculdade de Medicina da Universidade de São Paulo (FMUSP), São Paulo, Brazil; 4 Neuromuscular disorders Group, Child Neurology Unit, Hospital Universitari Vall d’Hebron, Vall d’Hebron Research Institute (VHIR), Barcelona, Spain; 5 University of British Columbia, Vancouver, British Columbia, Canada; 6 Department of Neurology, H. Houston Merritt Neuromuscular Research Center, Columbia University Medical Center, New York, NY, USA; 7 Molecular and Clinical Sciences Institute, St. George’s, University of London, Cranmer Terrace, London, UK; 8 Medical Genetics Research Centre, Shahid Sadoughi University of Medical Sciences, Yazd, Iran; 9 Department of Paediatric Neurology, Nottingham City Hospital, Nottingham University Hospitals NHS Trust, Hucknall Road, Nottingham, UK; 10 MRC Centre for Neuromuscular Diseases, National Hospital for Neurology and Neurosurgery, London, UK; 11 Dubowitz Neuromuscular Centre and MRC Centre for Neuromuscular Diseases, UCL Great Ormond Street Institute of Child Health, London, UK; 12 Manchester Centre for Genomic Medicine, St Mary’s Hospital, Manchester University NHS Foundation Trust, Health Innovation Manchester, Manchester, UK; 13 Department of Paediatric Neurology, Royal Preston Hospital, Preston, UK; 14 Department of Clinical Neurophysiology, Hospital Universitari Vall d’Hebron, Barcelona Spain; 15 Friedrich-Baur-Institute at the Department of Neurology, University Hospital LMU Munich, Munich, Germany; 16 Institute of Genetic Medicine, Central Parkway, Newcastle upon Tyne, UK; 17 Department of Neuropathology, John Radcliffe Hospital NHS Foundation Trust, Oxford, UK; 18 Department of Clinical Neurophysiology, John Radcliffe Hospital NHS Foundation Trust, Oxford, UK; 19 Department of Paediatric Neurology, John Radcliffe Hospital NHS Foundation Trust, Oxford, UK; 20 Department of Neuropediatrics and Muscle Disorders, Medical Center-University of Freiburg, Faculty of Medicine, Freiburg, Germany; 21 Centro Nacional de Análisis Genómico (CNAG-CRG), Center for Genomic Regulation, Barcelona Institute of Science and Technology (BIST), Barcelona, Spain; 22 Children’s Hospital of Eastern Ontario Research Institute, University of Ottawa, Ottawa, Canada; 23 Division of Neurology, Department of Medicine, The Ottawa Hospital, Ottawa, Canada

**Keywords:** congenital myasthenic syndromes, COL13A1, synaptic basal lamina, salbutamol, 3,4-diaminopyridine

## Abstract

Next generation sequencing techniques were recently used to show mutations in *COL13A1* cause synaptic basal lamina-associated congenital myasthenic syndrome type 19. Animal studies showed COL13A1, a synaptic extracellular-matrix protein, is involved in the formation and maintenance of the neuromuscular synapse that appears independent of the Agrin-LRP4-MuSK-DOK7 acetylcholine receptor clustering pathway. Here, we report the phenotypic spectrum of 16 patients from 11 kinships harbouring homozygous or heteroallelic mutations in *COL13A1.* Clinical presentation was mostly at birth with hypotonia and breathing and feeding difficulties often requiring ventilation and artificial feeding. Respiratory crisis related to recurrent apnoeas, sometimes triggered by chest infections, were common early in life but resolved over time. The predominant pattern of muscle weakness included bilateral ptosis (non-fatigable in adulthood), myopathic facies and marked axial weakness, especially of neck flexion, while limb muscles were less involved. Other features included facial dysmorphism, skeletal abnormalities and mild learning difficulties. All patients tested had results consistent with abnormal neuromuscular transmission. Muscle biopsies were within normal limits or showed non-specific changes. Muscle MRI and serum creatine kinase levels were normal. In keeping with *COL13A1* mutations affecting both synaptic structure and presynaptic function, treatment with 3,4-diaminopyridine and salbutamol resulted in motor and respiratory function improvement. In non-treated cases, disease severity and muscle strength improved gradually over time and several adults recovered normal muscle strength in the limbs. In summary, patients with *COL13A1* mutations present mostly with severe early-onset myasthenic syndrome with feeding and breathing difficulties. Axial weakness is greater than limb weakness. Disease course improves gradually over time, which could be consistent with the less prominent role of COL13A1 once the neuromuscular junction is mature. This report emphasizes the role of collagens at the human muscle endplate and should facilitate the recognition of this disorder, which can benefit from pharmacological treatment.

## Teaser

Mutations in *COL13A1* cause synaptic basal lamina-associated congenital myasthenic syndrome type 19. Rodriguez Cruz *et al.* describe the clinical and genetic spectrum associated with *COL13A1* mutations, and the key clinical features to look out for.

## Introduction

The use of next generation sequencing (NGS) in clinical diagnosis is allowing the identification of novel disease genes in neuromuscular disorders (Taylor *et al.*, 2015). This technology has been crucial to expand the genetic spectrum of the congenital myasthenic syndromes (CMS), which currently exceeds 30 genes (Rodríguez Cruz *et al.*, 2018). Causative genes encode for proteins that are essential for the integrity of neuromuscular transmission. The most common classification of CMS relies on the location of the encoded protein into presynaptic, synaptic or basal lamina-associated and postsynaptic syndromes. All subtypes of CMS share the feature of fatigable muscle weakness but age of onset, presenting symptoms, distribution of weakness, and response to treatment vary depending on the molecular mechanism that results from the underlying genetic defect.

**Figure 1:**
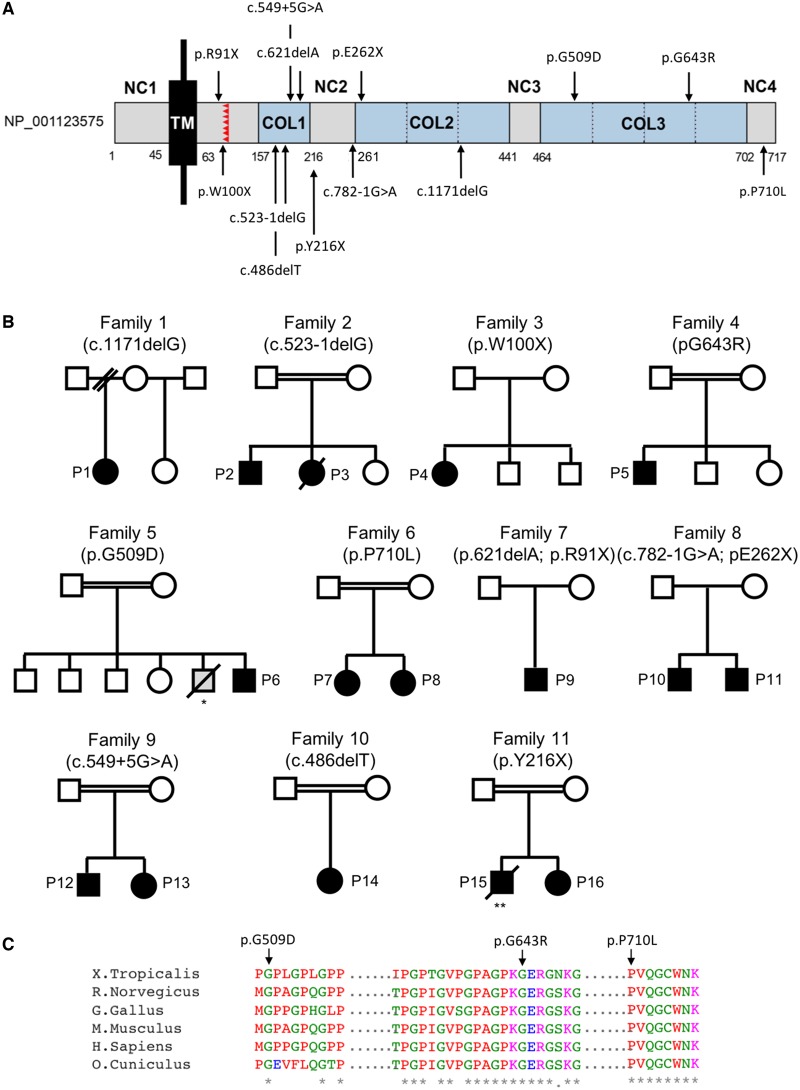
**Schematic representation of full-length COL13A1, location of genetic variants and pedigrees of families reported.** (**A**) COL13A1_001 (NP_001123575) consist of a short intracellular domain (NC1), a single transmembrane domain (TM), and the extracellular region with three collagenous domains (COL1–3) separated by short non-collagenous domains (NC2–3). The proprotease recognition site is labelled in red. Numbers indicate the amino acid residues composing each domain and the location of the pathogenic variants identified. Because COL13A1 undergoes complex alternative splicing, primary structures can vary. (**B**) Pedigrees of families included in this report. *An elder sibling from Patient 5 with similar symptoms died at an early age but genetic confirmation was not available. **Patient 15 was initially diagnosed with congenital insensitivity to pain with anhidrosis at the age of 1 year due to a homozygous *NTRK1* mutation (Mardy *et al.*, 1999). Patient 16, heterozygous for the *NTRK1* variant, was diagnosed with a myasthenic syndrome. This prompted the re-evaluation of the proband in whom a myasthenic pattern of muscle activation was found on neurophysiological studies (Raspall-Chaure *et al.*, 2005). Circles = female; squares = male; filled symbols = affected; open symbols = unaffected; strikethrough = deceased. (**C**) Protein alignments were performed using the Clustal Omega multiple sequence alignment program (https://www.ebi.ac.uk/Tools/msa/clustalo/).

Mutations in *COL13A1* were recently identified as the cause of autosomal recessive synaptic basal lamina-associated CMS type 19 (Logan *et al.*, 2015). *COL13A1* encodes the collagen type XIII alpha1 chain (COL13A1), which is a single-pass type II transmembrane protein made of a short intracellular domain, a single transmembrane domain, and a triple-helical collagenous ectodomain (Fig. 1A) (Pihlajaniemi and Tamminen, 1990). Unlike most of the collagens, COL13A1 is anchored to the plasma membrane by a hydrophobic transmembrane segment (Hägg *et al.*, 1998). The presence of a proprotease recognition site in the ectodomain allows the C-terminus to be proteolytically cleaved into a soluble form that is part of the basal lamina. Of note, *COL13A1* transcripts undergo complex alternative splicing (Pihlajaniemi and Tamminen, 1990).

The overall function of the *COL13A1* gene product is not well known, although mRNA expression has been detected at low levels in a wide range of tissues [EMBL-EBI Expression Atlas, https://www.ebi.ac.uk/gxa/home (Petryszak *et al.*, 2016)], suggesting a general role in the function of connective tissues such as cell-matrix and cell-cell interactions (Nykvist *et al.*, 2000; Tu *et al.*, 2002). This is also the case for muscle tissue although it should be noted that these studies did not take into account expression from sub-synaptic nuclei that are key in determining protein expression levels at the neuromuscular junction (Schaeffer *et al.*, 2001).

Studies using transgenic mice have shown that muscle-derived COL13A1 is essential for the maturation of the neuromuscular junction at both pre- and postsynaptic levels (Latvanlehto *et al.*, 2010; Härönen *et al.*, 2017). *Col13a1^−/−^* animals showed considerable presynaptic defects such as abnormal clustering of synaptic vesicles at the nerve terminal, reduced terminal complexity with defective nerve endings and terminal Schwann cells that were unable to cover the muscle endplates. The postsynaptic structures showed abnormal maturation with endplates that remained small, immature and fragmented compared to wild-type animals. In keeping with this, experimental studies on C2C12 muscle cell cultures have shown abnormal agrin-induced clustering of acetylcholine receptors (AChRs) with COL13A1 loss of function (Logan *et al.*, 2015). Interestingly, the deleterious effect on AChR clustering appears to be independent of any dramatic effect *in vivo* on key AChR-clustering-pathway proteins MuSK and DOK7 (Logan *et al.*, 2015). More recent studies in *Col13a1^−/−^* animals have shown that the disease tends to stabilize in adulthood once the neuromuscular junction is mature, suggesting that this is collagen is particularly relevant during development and early life (Zainul *et al.*, 2018).

Here we review in detail the mutational and clinical spectrum of disease associated with COL13A1-CMS in order to produce a detailed clinical picture that allows increased recognition of this disorder.

## Materials and methods

Next-generation and conventional Sanger sequencing were used to identify the underlying genetic mutations in *COL13A1* in 16 subjects from 11 different kinships. They all shared similar clinical features and had abnormalities in neurophysiological testing suggestive of abnormal neuromuscular transmission. The genetics of Cases 1–3 were previously reported by Logan *et al.* (2015) together with a short clinical description.

### Identification of COL13A1-CMS cases

A total of 16 patients (eight females) from 11 different kinships were included (Fig. 1B). Consanguinity was reported in seven families. Details on ethnicity are provided in Table 1.

**Table 1:** Clinical features of patients with CMS type 19

**Family Country Ethnicity**	**Family 1 UK WE**	**Family 2 UK Indian**		**Family 3 S. Africa WE**	**Family 4 UK Pakistani**	**Family 5 Qatar**	**Family 6 Iran**		**Family 7 Canada WE**	**Family 8 USA Bangladeshi**		**Family 9 Brazil WE**		**Family 10 Brazil WE**		**Family 11 Spain WE**
**ID**	**Pt 1**	**Pt 2**	**Pt 3**	**Pt 4**	**Pt 5**	**Pt 6**	**Pt 7**	**Pt 8**	**Pt 9**	**Pt 10**	**Pt 11**	**Pt 12**	**Pt 13**	**Pt 14**	**Pt 15^a^**	**Pt 16**
Consanguinity	N	Y	Y	N	Y	Y	Y	Y	N	N	N	Y	Y	Y	Y	Y
*COL13A1* mutations	c.1171delG (p.L392SfsX71)	c.523-1 delG	c.523-1 delG	c.300G>A (p.W100X)	c.1927G>C (p.G643R)	c.1526G>A (p.G509D)	c.2129C>T (p.P710L)	c.2129C>T (p.P710L)	c.621delA (p.G208EfsX15) c.271C>T (p.R91X)	c.782-1G>A c.784G>T (p.E262X)	c.782-1G>A c.784G>T (p.E262X)	c.549+ 5G>A	c.549+ 5G>A	c.486delT (p.G163 VfsX32)	c.648C>G (p.Y216X)	c.648C>G (p.Y216X)
Sex	F	M	F	F	M	M	F	F	M	M	M	M	F	F	M	F
Age current	6 y	27 y	Died 8 y	37 y	18 y	6 y	4 y	6 y	2 y	2 y	8 y	30 y	21 y	11 y	Died 20 y	22 y
Age assessed	5 m	24 y	5 y	37 y	13 y	5 y	2 m	1 y	18 m	3 m	5 y	29 y	18 y	8 y	20 y	22 y
Age at onset	Birth	Birth	Birth	1 y	Birth	Birth	Birth	Birth	Birth	Birth	Birth	Birth	Birth	Birth	Birth	Birth
Pregnancy	Normal	Normal	Normal	Normal	Normal	Normal	Normal	Normal	Normal	Normal	Normal	Normal	Normal	Normal	Normal	Normal
Presenting symptoms	BD, FD, hypotonia	Pt, BD, FD	BD, FD	MD, neck weakness	FD, hypotonia	BD, FD, RTI	BD, FD, pt	BD, FD, pt	BD, FD, hypotonia	BD, FD, hypotonia	BD, FD, hypotonia	BD, FD, pt, hypotonia	FD, pt, hypotonia	pt, hypotonia	FD, pt, hypotonia	BD, FD, pt, hypotonia
Ptosis	+++	++	+	++	++	+	++	+	+	+	+	++	++	++	+++	+++
Ophthalmoparesis	-	-	-/+	-/+	-	-/+	-	-	-	-	-	-	+	-	-	-
Facial weakness	++	+	+	+	+	+	+	+	+	++	++	+	+	+	+	+
Bulbar weakness	+++ (g)	-	++ (g)	-	+	+	+	+	+++ (g)	++ (g)	++ (g)	-	-	-	+	+ (g)
Axial weakness	+++	+	++	+++	+++	++	+	+	+++	+++	+++	++	++	+	++	++
Prox UULL	++	-	+	-	+	+	-	-	+	+	+	-	-	-	+	+
Prox LLLL	+	-	+	-	-	+	+	+	+	+	+	-	-	-	+	+
Distal UULL	+	-	+	+	+	-	-	-	+	+	+	-	-	-	-	-
Distal LLLL	+	-	+	-	-	-	-	-	+	+	+	-	-	-	-	-
Respiratory crisis	Y	N	Y	Y	N	Y	Y	N	Y	Y	Y	Y	N	N	N	Y
Ever required vent / trach	Y / N	N / N	Y / Y	Y / N	N / N	Y / N	Y / N	N / N	Y / Y	Y / N	Y / Y	Y / N	Y / N	N / N	N / N	Y / Y
Current use of NIV / trach	Y / N	N / N	Na	Y / N	N / N	N / N	N / N	N / N	Y / Y	Y / N	Y / Y	Y / N	Y / N	N / N	N / N	Y / N
Dysmorphic features	Y	Y	Y	Y	Y	Y	N	N	Y	N	Y	Y	Y	Y	Y	Y
Kyphosis / scoliosis	N / N	N / N	Y / N	N / Y	Y / Y	N / N	N / N	N / N	N / N	N / N	N / Y	N / Y	N / Y	N / N	N / Y	N / Y
Contractures	N	N	N	N	N	N	N	N	N	N	N	N	N	N	Y	N
Distal joint laxity	Y	N	Y	N	N	N	N	N	N	N	Y	Y	Y	N	-	-
Delayed motor milestones	Y	?	Y	Y	Y	Y	N	N	Y	Y	Y	Y	Y	Y	Y	Y
Learning difficulties	Y	Y	NK	N	Y	Y	N	N	NK	Y	Y	N	N	N	Y	N
Treatment	Sb, DAP, Py −ve	None	Py −ve, NIV	Sb, NIV	Py −ve, Sb (inh)	Sb, DAP	Sb, py	None	Sb, DAP	Sb, DAP	Sb (inh), py	Py −ve	Py −ve	Py −ve	-	Sb, DAP

BD = breathing difficulties; DAP = 3,4-DAP; FD = feeding difficulties; F = female; g = gastrostomy; inh = inhaler; M = male; MD = motor delay; N = no; NA = not applicable; NIV = non-invasive ventilation; NK = not known; pt = ptosis; py = pyridostigmine; RTI = respiratory tract infections; sb = salbutamol; WE = white European ancestry; Y = yes. ^a^Patient 15 was also diagnosed with congenital insensitivity to pain with anhidrosis due to homozygous NTRK1 mutations (Mardy *et al.*, 1999) while Patient 16 was genetically confirmed heterozygous.

### Genetic analysis

Genomic DNA was isolated from patients’ and parents’ blood by standard methods. Exome sequencing was carried out using the manufacturer’s specifications. Sanger sequencing was performed with primers covering exonic and flanking regions of *COL13A1.* Analysis of splicing variants was performed with Human Splice Finder 3.1 (Desmet *et al.*, 2009). Ethics approval for analysis of DNA and tissue samples was obtained (OXREC B: 04.OXB.017 and Oxfordshire REC C 09/h0606/74).

### Endplate studies

Fresh frozen muscle sections from Patient 1 were labelled with Alexa Fluor® 594 conjugated α-bungarotoxin (Life Technologies, Cat. No. B13423) and Alexa Fluor® 488-fasciculin (Life Technologies, special order) at 1 μg/ml for 1 h at 37°C to stain for AChRs and acetylcholinesterase (AChE), respectively. The presynaptic Schwann cell marker S100β was labelled using a mouse monoclonal anti-S100β antibody (Sigma, Cat. No. SAB1402349) and the corresponding fluorescently conjugated secondary antibody (Life Technologies, Cat. No. R37115). Then, sections were washed in PBS and fixed for 10 min in 3% paraformaldehyde at room temperature. Images from the muscle endplates were taken using a Zeiss LSM 510 inverted confocal microscope. Co-localization studies were performed using ImageJ software (Schindelin *et al.*, 2012).

### Data availability

The data that support the findings of this study are available from the corresponding author, upon reasonable request.

## Results

### Genetic analysis

Whole exome sequencing identified 13 *COL13A1* variants in 16 subjects from 11 different kinships identified by whole exome sequencing (Fig. 1A, B and Table 1). PCR amplification on genomic DNA and Sanger sequencing confirmed the mutations and segregation of *COL13A1* variants with disease. Ten variants were loss-of-function [nonsense (4), frameshift (3), and splice-site (3)] mutations and three were missense. Two siblings were homozygous for the c.523-1delG splice-site variant, which is predicted to allow splicing but lead to premature termination due to a single-base deletion in the coding sequence (p.Gly175Vfs*20) (Logan *et al.*, 2015). Two siblings were heterozygous for the c.782-1G>A splice-site variant, which is predicted to abolish the wild-type donor site and may lead to activation of an intronic cryptic acceptor site. In addition, two siblings were homozygous for the c.549+5G>A splice site variant, which is predicted to alter the wild-type donor site. Both splice variants were predicted to ‘most probably’ affect splicing by Human Splice Finder 3.1 software (Desmet *et al.*, 2009). The three missense variants identified (p.G509D, p.G643R and p.P710L) were located in the C-terminal domain of the protein and affect amino acids evolutionarily conserved across species (Fig. 1C). *In silico* analysis classified all missense variants as damaging by MutationTaster (Schwarz *et al.*, 2010), PolyPhen-2 (Adzhubei *et al.*, 2010) and the SIFT algorithm (Sim *et al.*, 2012) (Supplementary Table 1). None of the variants identified by whole exome sequencing were listed in Ensembl genome browser 94 [(EMBL-EBI, Cambridge, UK (URL: https://www.ensembl.org) (Dec 2018)] (Zerbino *et al.*, 2018) or in the 125 748 exome sequences and 15 708 whole-genome sequences from unrelated subjects of the Genome Aggregation Database [(gnomAD, Cambridge, MA (URL: http://gnomad.broadinstitute.org) (Dec 2018)] (Lek *et al.*, 2016) except for c.621delA;p.G208EfsX15 (Ensembl: allele frequency not provided), c.271C>T;p.R91X (Ensembl: 0.001 in ALSPAC cohort and 0.000 in TWINSUK cohort) and c.486delT;p.G163VfsX32 (0.000004035; gnomAD), which were classified as rare variants according to their allele frequencies displayed in brackets.

### Clinical features

#### Clinical presentation

Pregnancy was uneventful in all cases and normal foetal movements were reported. Clinical presentation was mostly at birth or shortly afterwards (median age at onset was 0 years, range 0–1 years) with varying degrees of feeding and breathing difficulties. Common features were, first, a number of cases (Patients 2, 5–8 and 12–13) presented with poor suck and weight loss in the neonatal period, and some associated additional recurrent episodes of respiratory dysfunction. This subgroup did not require overall prolonged artificial feeding or ventilation, although we note that Patient 5 was not started on solid food until the age of 3. Second, a more severe group of patients had marked breathing and feeding difficulties requiring long-term ventilation or tracheostomy and artificial feeding from birth (Patients 1, 3, 9–11, 15 and 16). For instance, Patient 1 had severe bulbar weakness and recurrent apnoeas related to diaphragmatic weakness requiring non-invasive ventilation and long-term gastrostomy. In the same way, Patient 9 was apnoeic from birth and had significant bulbar weakness with fatigue on crying and subsequent evidence of failed extubations due to diaphragmatic weakness and respiratory failure. Finally, on the less severe side of the clinical spectrum, Patient 14 was only noted to have mild hypotonia and bilateral ptosis in the first weeks of life, and Patient 4 presented with difficulties to crawl at the age of 1 year, associated with marked neck muscle weakness requiring a neck brace from the ages of 2 to 3 years. Overall, bilateral ptosis (8/16) and generalized hypotonia (10/16) from birth were reported in approximately half of the subjects.

#### Pattern of muscle weakness

**Figure 2:**
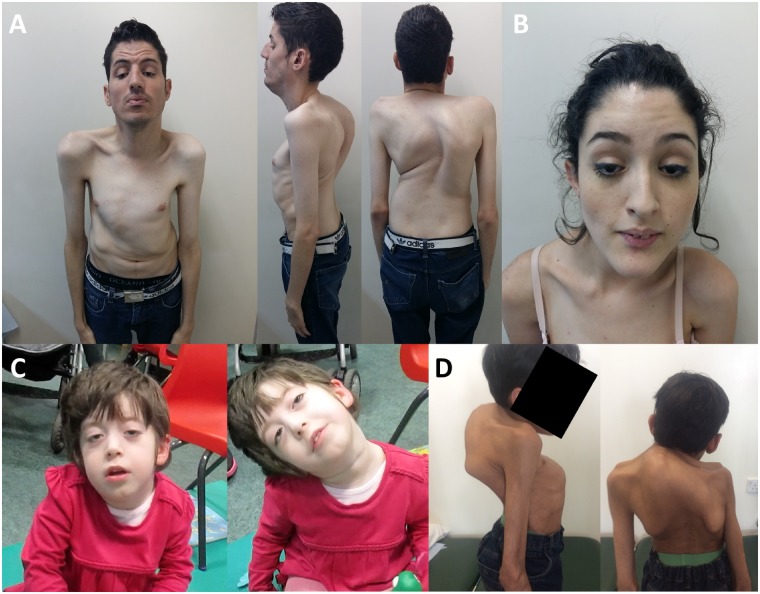
**Clinical features of patients with *COL13A1* mutations.** (**A** and **B**) Presence of bilateral ptosis and dysmorphic features including elongated face, micrognathia and low-set ears (Patients 12 and 13). (**C**) Marked weakness in neck flexors and truncal muscles with poor head control (Patient 1). (**D**) Severe scoliosis and associated thoracic kyphosis with restricted lung capacity (Patient 5).

The predominant pattern of muscle weakness included bilateral ptosis with differing degrees of severity, myopathic facies and marked axial weakness, especially in neck flexors and trunk muscles (Fig. 2A and B). Limb muscles were generally less severely affected. Interestingly, in adult cases, ptosis was mainly described as non-fluctuating and was usually non-fatigable on examination. Eye movements were fully preserved except for mild restriction of upgaze in a few (Patients 3, 4, 6 and 13) and no double vision was reported. The presence of a squint was only noted in Patient 6. All cases had myopathic facies with mild to moderate facial weakness involving eye closure and muscles of the lower face. There was marked weakness in neck flexors and extensors and truncal muscles, with a number of patients experiencing poor head control (Fig. 2C) and scoliosis (Fig. 2D). Weakness in proximal limbs was generally present early in life but improved gradually over time, with some patients having no detectable weakness in adulthood (Patients 2, 4, 12 and 13). Mild distal weakness in upper and lower limbs was noted in half of the cases.

#### Respiratory function

Respiratory function was compromised in the majority of patients, though with differing degrees of severity, including four patients requiring long-term tracheostomy soon after birth. Episodes of respiratory deterioration were frequent early in life, and ventilator support was often needed. Respiratory crisis were either triggered by chest infections or occurred spontaneously, and some patients had associated bibasilar atelectasis probably related to diaphragmatic weakness. Overall, the prognosis over time was favourable with a reduction in the frequency of respiratory events, although they still occurred in some adult cases with reduced vital capacity and chest abnormalities: Patient 4 had two respiratory crises triggered by chest infections at the age of 32 years following a long period of stability of more than a decade. Patients 12 and 13 suffered from daily somnolence in adulthood and were diagnosed with obstructive sleep apnoea by polysomnography and subsequently started on nocturnal non-invasive ventilation.

#### Additional clinical features

Dysmorphic features were noted in all cases except Patients 7, 8 and 10. These included elongated face, low-set ears, micrognathia and high-arched palate (Fig. 2). In addition, some patients (Patients 1–3, 5, 6 and 15) had prominent skeletal abnormalities, especially of the chest (Fig. 2A and D), with the presence of pectus carinatum from early life. Patients 4, 5, 12, 13 and 16 developed moderate to severe scoliosis with associated thoracic kyphosis and restricted lung capacity in some cases (Fig. 2D). Spinal fusion surgery was performed in Patients 4 and 16 at the age of 8 and 13 years, respectively. Mild spinal rigidity was present in Patients 2 and 3 but no distal or proximal contractures were noted in any patient. Joint laxity was not a striking feature and was only noted mild distally in Patients 1, 3 and 11–13. An overall thin appearance with generalized reduced muscle bulk was reported in the records of Patients 2, 4, 5, 12 and 13.

Mild learning difficulties were present in Patients 1, 2, 5, 6 and 10, while Patient 11 had severe cognitive delay with autism spectrum disorder and self-injurious behaviour. At the age of 8 years, he is able to follow simple commands and has ∼30–40 words in his vocabulary. Patient 15 had moderate cognitive delay and self-mutilation behaviour secondary to congenital insensitivity to pain with anhidrosis due to a homozygous mutation in *NTRK1* (Mardy *et al.*, 1999). Skin abnormalities were only noted in Patient 1 with mild keratosis pilaris. There were no signs suggestive of skin hyperextensibility or hypertrophic scarfs. Patient 3 had a combined hiatus and diaphragmatic hernia that worsened her respiratory function. Patient 5 had delayed recovery from general anaesthesia at age 2 months following surgery for a unilateral inguinal hernia.

#### Response to treatment

While there was no clear response to cholinesterase inhibitors, treatment with 3,4-diaminopyridine (3,4-DAP, 0.3–0.9 mg/kg/day) and salbutamol (0.05–0.56 mg/kg/day) resulted in improved motor and respiratory function. Treatment with 3,4-DAP (0.3 mg/kg/day) and salbutamol (0.56 mg/kg/day) was effective in Patient 1, leading to better head control, improved unassisted sitting and reduced requirement for non-invasive ventilation. Previous treatment with pyridostigmine (up to 6 mg/kg/day) was only transiently beneficial. Patient 2 did not respond to treatment with pyridostigmine up to 6 mg/kg/day in childhood. Further treatment was not attempted because of the normalization of his muscle strength in adulthood. Patient 3 was not initiated on pyridostigmine because of parental choice at the time of clinical diagnosis. Patient 4 had no further respiratory crisis to date on treatment with salbutamol (0.17 mg/kg/day) although neck weakness continues to be marked. Patient 5 was already on treatment with salbutamol inhaler for asthma and therefore further treatment was not advised. Patient 6 improved his axial strength and fatigue levels on treatment with 3,4-DAP (0.9 mg/kg/day) and salbutamol (0.2 mg/kg/day). Patient 7 was treated with pyridostigmine and salbutamol inhaler (because of asthma) with apparent improvement in her ptosis. Patient 8 did not receive pharmacological treatment for her mild myasthenic symptoms. Treatment with salbutamol (up to 0.2 mg/kg/day) in Patient 9 was initially useful to wean from invasive to non-invasive ventilation. Subsequent introduction of 3,4-DAP (0.3 mg/kg/day) helped to improve his head control and limb strength and reduce his fatigue when crying. Patient 10 was started on 3,4-DAP (0.3 mg/kg/day) and albuterol (0.56 mg/kg/day) at 3 months of age with overall improvement in motor function and gain of antigravity power in limbs and ability to sit without support. The response to treatment in Patient 11 has not been assessed. Patients 12–14 did not respond to treatment with pyridostigmine. Patient 16 had some improvement on 3,4-DAP, which was helpful for decannulation and switching to non-invasive ventilation and removal of gastrostomy at age 4 years. Subsequent addition of salbutamol (0.15 mg/kg/day) resulted in clear respiratory improvement (vital capacity increased from 20% to 40% of the predicted value) with no respiratory crisis in the last 5 years.

#### Course of disease

**Figure 3:**
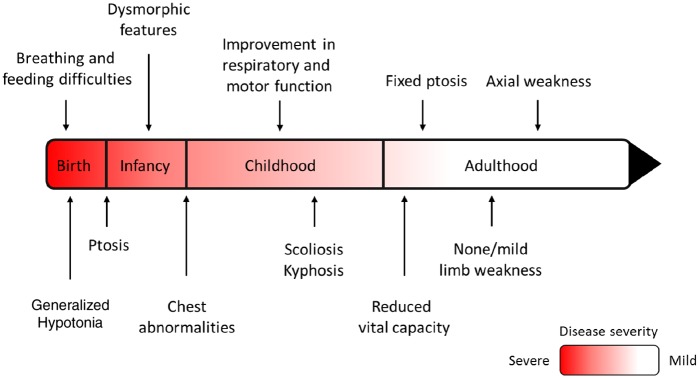
**Course of disease in patients with COL13A1-CMS.** The proposed course of disease in COL13A1-CMS based on the observations made from the cases reported in this study. Axial weakness remained severe throughout the disease course while limb weakness and bulbar weakness improved over time. The respiratory function also improved with time but some adult patients had respiratory crisis or needed non-invasive ventilation most likely due to morphological abnormalities of the chest and the spine causing reduced vital capacity.

Most patients improved over time with regards to their motor and respiratory function, including a decrease in the frequency of respiratory events (Fig. 3). This was obvious in the adults (Patients 2, 4, 5, 12, 13 and 16) where examination showed absent or minimal weakness in limb muscles despite some of them not being on treatment at the time of diagnosis. By contrast, axial weakness persisted in adulthood with most cases experiencing ongoing moderate to severe neck weakness and poor head control. Respiratory function was also impaired in adult patients with reduced vital capacity, scoliosis and morphological abnormalities of the chest (Patients 4, 5, 12 and 13). All adult patients were fully ambulant although Patient 5 could walk only up to 400 m before he became fatigued and short of breath. The milder paediatric cases (Patients 6–8 and 14) gradually improved with age although remain affected with bilateral ptosis and mild to moderate axial weakness, but free of respiratory events. The more severe paediatric cases with onset of symptoms at birth (Patients 1, 9 and 10) continue to make good progress except for Patient 11 who gained the ability to cruise at age 5 years but subsequently lost it and since then has been wheelchair-dependent. Patient 1 at 5 years of age is currently able to walk independently around the house but uses a wheelchair for longer distances. She is largely percutaneous endoscopic gastrostomy (PEG)-fed and uses non-invasive ventilation at night, although she has remained free of respiratory crisis since salbutamol was initiated at 2 years of age. Axial weakness is still present with sub-gravity neck flexion strength and poor head control. Patient 9 remains PEG-fed and ventilated via tracheostomy at age 2 years. However, there has been a reduction in fatigue and progress in his abilities with less frequent need for suctioning, acquisition of the ability to sit and improvement in head control and limbs strength. Patient 10 continues to have poor head control with inability to stand without support at age 23 months. He requires nocturnal non-invasive ventilation and is fully PEG-fed. Patient 3 died at the age of 8 years from chronic respiratory failure attributed to muscle weakness and diaphragmatic hernia, and Patient 15 died at the age of 20 years from a choking episode.

### Investigations

#### Muscle biopsy

**Figure 4:**
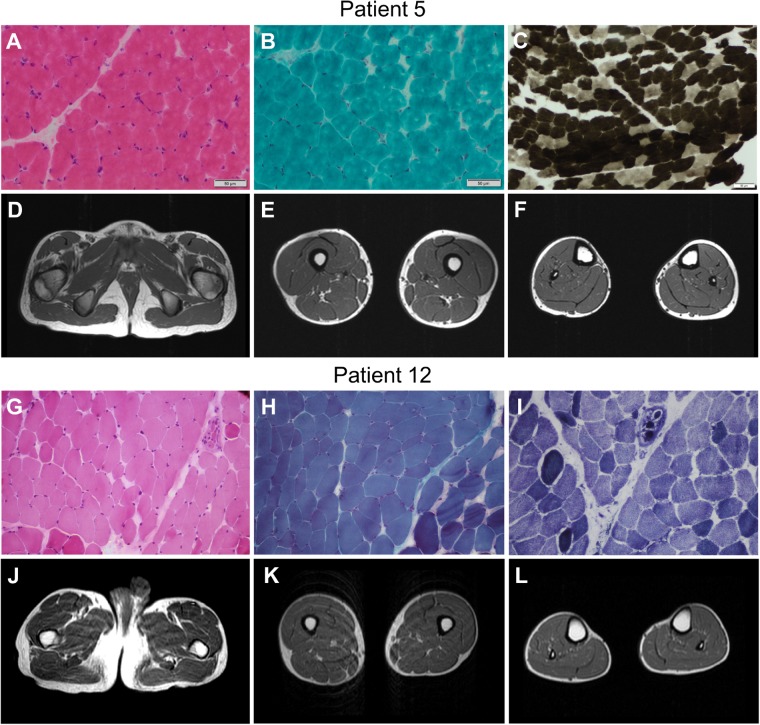
**Clinical investigations of patients with *COL13A1* mutations.** Muscle biopsy from the quadriceps muscle in Patient 5 at the age of 5 years showed mild changes on haematoxylin and eosin stain (**A**) and modified Gomori trichrome (**B**), and type 1 fibre predominance on the ATPase 4.3 enzyme histochemical stain (**C**). Muscle MRI of his pelvis and lower limbs at the age of 13 years was normal (**D**–**F**). Muscle biopsy from the biceps brachialis in Patient 12 at the age of 17 years showed mild variability of fibre size on haematoxylin and eosin stain (**G**). Modified Gomori trichrome staining showed a mild increase in perimysial connective tissue (**H**) and nicotinamide adenine dinucleotide tetrazolium reductase (NADH-TR) stain showed mild disruption of the myofibrillar architecture (**I**). Muscle MRI of his pelvis and lower limbs showed no abnormalities (**J**–**L**).

Nine patients underwent muscle biopsy (age range: 6 months–17 years), which was described as normal or showed mild non-specific changes (Table 2 and Fig. 4). These included: mild variation in fibre size (Patients 1, 5, 6 and 12; Fig. 4A and G) and the presence of a few internal nuclei (Patient 1); mild increase in connective tissue (Patients 12 and 15); mild type 1 fibre predominance (Patient 5; Fig. 4C); and mild changes in oxidative staining with some fibres giving a moth-eaten (Patient 12; Fig. 4I) or a halo-like appearance (Patient 1). Occasional peripheral vacant vacuole-like areas with haematoxylin and eosin stain and several hypercontracted fibres with Gömöri trichrome stain were seen in Patient 1 although an artefactual effect cannot be ruled out (Logan *et al.*, 2015).

**Table 2:** Clinical investigations of patients with CMS type 19

	Family 1	Family 2		Family 3	Family 4	Family 5	Family 6	
Affected individual	**Patient 1**	**Patient 2**	**Patient 3**	**Patient 4**	**Patient 5**	**Patient 6**	**Patient 7**	**Patient 8**
Vital capacity, l, (% predicted)	–	2.88 (62)	–	1.08 (29)	0.75 (23)	–	–	–
Abnormal RNS decrement (>10%)	Y	Y	–	Y	N	N	Y	Y
Muscle	ADM, FHB	Anconeous	–	Trapezius	FCU	NA	ADM, FHB	ADM, FHB
Abnormal SFEMG	Y	Y	–	Y	Y	Y	–	–
Muscle	OO	EDC	–	OO	OO	OO	–	–
MCD, µs	133.3	69.8	–	173.8	68.7	75.0	–	–
Increased jitter	Y	Y	–	Y	Y	NA	–	–
Blocking	Y	N	–	N	Y	NA	–	–
Muscle biopsy (age)	Y (6 m)	–	Y (1 y)	Y (3 y)	Y (5 y)	Y (1 y)	–	–
Muscle	Quadriceps	–	Quadriceps	NA	Quadriceps	Quadriceps	–	–
Result	Non-specific	–	Normal	NA	Non-specific	Non-specific	–	–
Muscle MRI	Normal	–	–	–	Normal	–	–	–
CK (IU/l)	35	–	–	212	141	Normal^a^	Normal^a^	Normal^a^
Brain MRI	–	–	–	–	Normal	–	–	–

	**Family 7**	**Family 8**		**Family 9**		**Family 10**	**Family 11**	
Affected individual	**Patient 9**	**Patient 10**	**Patient 11**	**Patient 12**	**Patient 13**	**Patient 14**	**Patient 15**	**Patient 16**
Vital capacity, l, (% predicted)	–	–	–	1.43 (29%)	^b^	–	0.65 (14%)^c^	0.55 (23%)
Abnormal RNS decrement (>10%)	–	–	Y	N	N	Y	Y	Y
Muscle	–	–	Na	ADM, deltoid, TA, nasalis	ADM, deltoid, TA, nasalis	EDC, deltoid,	ADM	ADM
Abnormal SFEMG	–	–	–	Y	Y	–	Y	Y
Muscle	–	–	–	OO	OO	–	EDC	EDC
MCD (µs)	–	–	–	73	76	–	96	50
Increased jitter	–	–	–	Y	Y	–	Y	Y
Blocking	–	–	–	NA	NA	–	NA	Y
Muscle biopsy (age)	–	–	Y (8 m)	Y (17 y)	–	–	Y (6 m)	Y (15 y)
Muscle	–	–	Deltoid	Biceps brachialis	–	–	Quadriceps	Quadriceps
Result	–	–	Non-specific	Non-specific	–	–	Non-specific	Non-specific
Muscle MRI	–	–	–	Normal	Paraspinal, atrophy	–	–	Paraspinal, atrophy
CK (IU/l)	Normal	900 → 47	81	63	45	68	100	41
Brain MRI	Normal	Normal	Normal	–	–	–	Normal	Normal

ADM = abductor minimi digiti; CK = creatine kinase; FCU = flexor carpi ulnaris; FHB = flexor hallucis brevis; m = months; N = no; NA = not available; OO = orbicularis oculi; QMG = quantitative myasthenia gravis score; RNS = repetitive nerve stimulation; SFEMG = single-fibre EMG; WES = whole exome sequencing; y = years; Y = yes.

^a^Specific values are not available but recorded as normal in the patients’ records.

^b^Specific value not available but reported as ‘similar to sibling’ in the patient’s records.

^c^Patient was not fully collaborative during the test.

#### Neurophysiology

All patients tested had results consistent with abnormal neuromuscular transmission (Table 2). Repetitive nerve stimulation at 3 Hz showed the presence of significant decrement in 9 of 13 patients in proximal and/or distal muscles of the upper limbs. Frequency dependent decrement was not routinely assessed. No repetitive discharges or increment to volitional contraction was observed. Single fibre electromyography (SFEMG) showed increased jitter or blocking in all nine patients tested. Nerve conduction studies were normal whenever performed. Needle EMG examination showed additional myopathic features in Patients 5, 6 and 15.

#### Other investigations

Serum creatine kinase levels were normal in all cases. Patient 10 initially had raised creatine kinase levels in the neonatal period at 900 IU/l but values subsequently normalized to 47 IU/l. Brain MRI and muscle MRI of the lower limbs were reported as normal in all cases performed (Fig. 4D–F and J–L). Whole body muscle MRI, conducted in Patients 13 and 16 at the age of 21 and 15 years, respectively, showed changes suggestive of atrophy and moderate fatty replacement in the paraspinal muscles, although we note Patient 16 had spinal fusion surgery at age 13.

#### Endplate studies

**Figure 5:**
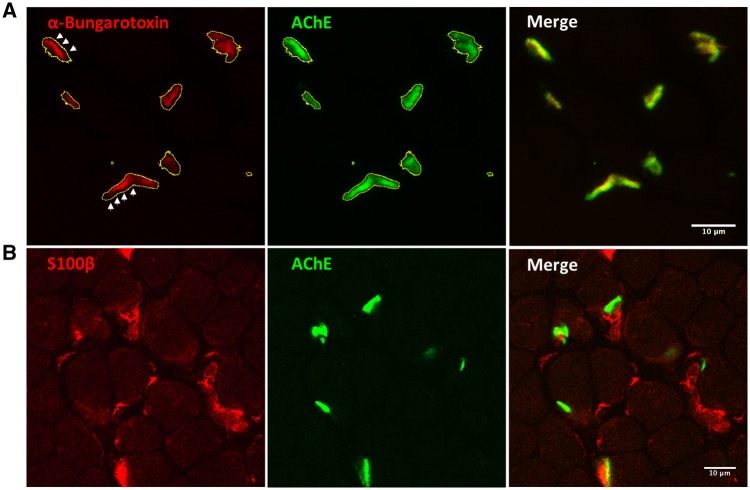
**Muscle endplate studies.** (**A**) Muscle biopsy from quadriceps femoris in Patient 1 at age 6 months were labelled with Alexa Fluor® 488**-**fasciculin and Alexa Fluor**®** 594**-**α-bungarotoxin and analysed using confocal microscopy and ImageJ software. The contour of the motor endplates corresponding to the fasciculine staining (green) was selected (yellow line) and then applied to the red channel. As shown by the arrows, the α-bungarotoxin staining (red) did not completely fill the selection area. (**B**) Staining for the presynaptic marker S100β showed terminal Schwann cells reaching the muscle endplates. It appeared that the presynaptic marker S100β did not fully cover some of the muscle endplates, although an age-matched control muscle biopsy to compare with was not available.

Co-localization studies in muscle endplates showed positive expression of AChRs and AChE but lack of complete overlap between the two fluorescent dyes with the contour of the acetylcholinesterase staining (green) exceeding the limits of the α-bungarotoxin staining (red) (Fig. 5A). Staining for the presynaptic marker S100β labelled terminal Schwann cells reaching the muscle endplates (Fig. 5B).

## Discussion

We describe the clinical spectrum of disease associated with *COL13A1* mutations and report a series of novel pathogenic mutations in this recently described CMS causative gene. Patients with *COL13A1* mutations underlie a myasthenic syndrome characterized by early onset muscle weakness with predominantly feeding and breathing difficulties often requiring ventilation and artificial feeding. The pattern of muscle weakness is predominantly axial with marked bulbar, neck and truncal weakness rather than appendicular. Scoliosis can be severe and therefore careful monitoring of the spinal curvature is recommended. Patients improve on treatment with 3,4-DAP and salbutamol with regards to their motor and respiratory function, whereas pyridostigmine was not beneficial. Disease severity improves gradually over time with reduced frequency of respiratory events with age and some patients having no or only mild muscle weakness on examination in adulthood.

Collagens are important components of the synaptic basal lamina, a specialized form of extracellular matrix that lies in the intersynaptic space and is essential for the neuromuscular junction architecture and function (Patton, 2003). Additional elements include laminins, heparan sulphate proteoglycans (muscle agrin and perlecan) and nidogens (Shi *et al.*, 2012). For many years, mutations in *COLQ* encoding the collagen-like tail subunit of asymmetric AChE were the only identified subtype of synaptic basal lamina-associated CMS (Ohno *et al.*, 1998). This has been expanded with the report of CMS due to *LAMB2* (Maselli *et al.*, 2009), *LAMA5* (Maselli *et al.*, 2017) and *COL13A1* mutations (Logan *et al.*, 2015), which has helped to increase our understanding on the contribution of the synaptic basal lamina to the organization of the neuromuscular synapse.

COL13A1-disease shares with other myasthenic syndromes the presence of fatigable muscle weakness and abnormal neurophysiology with decremental response to repetitive nerve stimulation and/or abnormal jitter on SFEMG. More specific features include facial dysmorphism, skeletal abnormalities of the chest, and a lack of beneficial response to cholinesterase inhibitors (Supplementary Table 2). Of interest, we note that similar facial and skeletal features have been reported in patients with King-Denborough syndrome (King and Denborough, 1973) although these are overall non-specific features and therefore can be found in other syndromes. King-Denborough syndrome is a rare condition characterized by dysmorphic features including ptosis, skeletal abnormalities, myopathy and malignant hyperthermia susceptibility, although the latter is not always present (Dowling *et al.*, 2011). The cause of King-Denborough syndrome is not fully understood, although some cases have been attributed to mutations in *RYR1* (D’Archy *et al.*, 2008).

Unlike other CMS, ptosis in patients with COL13A1-CMS is non-fluctuating and non-fatigable on examination in adulthood, compared to an early age. We could speculate that this might indicate a concomitant myopathic process impairing the levator palpebrae superioris function. We note that needle EMG examination showed myopathic changes in several patients, although creatine kinase levels, muscle biopsy and muscle MRI studies were overall normal, which is consistent with a myasthenic syndrome considering the degree of muscle weakness seen in the patients. Endplate studies carried out in the muscle biopsy of Patient 1 showed the incomplete overlap between acetylcholine receptors and acetylcholinesterase staining. The significance of this finding is unclear but we note that this has also been reported in the *Col13a1*^−/−^ animal model (Härönen *et al.*, 2017).

In general, CMS type 19 lies on the severe side of the CMS spectrum, with a meaningful proportion of patients having life-threatening feeding and breathing difficulties early in life. However, a small number of patients fall on the mild side of the spectrum (Patients 7 and 14) with minimal weakness and lack of respiratory events, which reflects a broad clinical spectrum. Phenotype-genotype correlation suggests that patients with missense mutations may have milder symptoms compared to those harbouring loss-of-function mutations. In keeping with this, none of the four patients reported with missense mutations required tracheostomy or artificial feeding, although Patient 5 has severe chest abnormalities and reduced vital capacity in adulthood.

The nature of the respiratory issues early in life is not clear, and it seems not to be correlated with limb weakness. The observation that some patients developed bibasilar atelectasis during the respiratory episodes points to diaphragmatic weakness, although weakness of accessory respiratory muscles and stiffness of the rib cage could also play a role. Other factors that seem to influence patients’ respiratory outcome in adulthood are the spine and chest abnormalities that may lead to restrictive lung disease and low vital capacity. Therefore, spinal curvature should be monitored periodically from early childhood.

Evaluation in adulthood suggests that patients with CMS type 19 improve gradually over time with regard to their respiratory and motor function, although some adult patients can also present with ongoing respiratory problems if their vital capacity is compromised. Recent experimental studies in *Col13a1^−/−^* animals have shown that disease severity stabilizes in adult mice once the neuromuscular junctions have matured (Zainul *et al.*, 2018). These findings suggest that this subtype of CMS could be particularly severe early in life due to the more prominent role of COL13A1 in the formation and maturation of the muscle endplate. We have not observed long-term fluctuations as reported in patients with *DOK7* mutations (Muller *et al.*, 2007).

Patients with *COL13A1* mutations do not appear to respond to treatment with pyridostigmine, but 3,4-DAP and salbutamol have been beneficial in improving motor and respiratory function. 3,4-DAP acts by blocking potassium channels at the presynaptic terminal and expands the duration of acetylcholine release, which would help compensate for the presynaptic abnormalities derived from the COL13A1 loss. The molecular mechanism of salbutamol at the neuromuscular junction is still not fully understood but recent insight supports an effect on maintenance of synaptic integrity (Clausen *et al.*, 2018; Mcmacken *et al.*, 2018). This suggests that β2-adrenergic agonists could work by compensating for the postsynaptic abnormalities and lack of endplate maturation derived from the loss of COL13A1 function. Early introduction of β2-adrenergic agonists could prove helpful to stabilize respiratory function and reduce the number of respiratory events, as observed in some of the cases reported here. The lack of response to pyridostigmine and the robust alpha-bungarotoxin endplate staining argue against a deficiency of endplate AChR.

RNA expression studies have shown that COL13A1 is widely expressed in different tissues. Therefore, it is unclear why *COL13A1* mutations primarily affect the neuromuscular junction although immunostaining does shows that COL13A1 is highly concentrated at the neuromuscular junction. A similar phenomenon occurs in patients with CMS and mutations within the *N*-glycosylation pathway, which is ubiquitously expressed (Belaya *et al.*, 2012; Cossins *et al.*, 2013; Zoltowska *et al.*, 2013). In connexion with this, several patients with *COL13A1* mutations had mild learning difficulties and one suffered from severe mental retardation within the autistic spectrum. It is possible that the cognitive impairment could be attributed to the loss of COL13A1 expressed in the brain (Uhlén *et al.*, 2015). However, additional contributing factors may include consanguinity, which is a well-known risk factor for genetic disorders that present with intellectual disability, and respiratory crisis early in life. The latter could result in hypoxic changes in the CNS, although brain MRI did not identify any structural abnormalities in the cases available. Finally, although less likely, cognitive deficits are relatively common in the general population, and their co-occurrence with COL13A1-CMS might be incidental. Future studies using standardized neuropsychological tests will be helpful to understand whether there is a specific defective pattern in cognition

In conclusion, this study expands the clinical and genetic spectrum of *COL13A1* disease and highlights the importance of collagens at the neuromuscular junction. The detailed description of clinical and complementary features of patients with CMS type 19 should facilitate the recognition and appropriate treatment of patients with this condition. This work also highlights the increasingly important role of next generation sequencing in routine clinical practice for reaching a definite genetic diagnosis.

## Supplementary Material

awz107_Supplementary_Tables

## Abbreviations

3,4-DAP: 3,4-diaminopyridine
CMS: congenital myasthenic syndromes
