# Supplementary material for: The clinical spectrum of the congenital myasthenic syndrome resulting from COL13A1 mutations
Source: Brain. 2019 May 13;142(6):1547–60. doi: 10.1093/brain/awz107 (PMC6752227; doi:10.1093/brain/awz107)
Supplement: awz107_Supplementary_Tables [file awz107_supplementary_tables.pdf]

Supplementary table 1: in-silico prediction of pathogenicity of *COL13A1* missense variants

COL13A1 variant	Polyphen-2 (score)	Mutation Taster (score)	SIFT algorithm
NM_001130103:c.1526G>A:p.G509D	Probable damaging (1.000)	Disease causing (0.999)	Damaging
NM_001130103:c.1927G>C:p.G643R	Probable damaging (1.000)	Disease causing (0.999)	Damaging
NM_001130103:c.2129C>T:p.P710L	Probable damaging (1.000)	Disease causing (0.999)	Damaging

Supplementary table 2: comparison of COL13A1 clinical features with most common CMS subtypes

	**COL13A1-CMS**	**AChR-deficiency**	**COLQ-CMS**	**DOK7-CMS**	**Glycosylation-CMS**	**Rapsyn-CMS**	**Slow channel syndrome**
Onset	birth	birth-infancy	birth-childhood (variable)	childhood (variable)	childhood-adulthood	birth (variable)	variable
Eye movements	normal	restricted	normal to moderately impaired	normal or mildly impaired	normal	normal or mildly impaired	variable
Ptosis	non-fatigable in adulthood	fatigable	fatigable	fatigable	none	fatigable	fatigable
Dysmorphic features	common	rare	rare	rare	none	common	none
Skeletal abnormalities	chest scoliosis	none	scoliosis hyperlordosis	scoliosis hyperlordosis	none	none	scoliosis in severe cases
Main pattern of muscle weakness	predominantly axial	facial and generalised weakness	facial and proximal weakness	facial and proximal weakness	predominantly proximal	generalised weakness	cervical and distal weakness
Characteristic features	neck weakness<br>Fixed ptosis<br>barrel chest<br>early respiratory problems	severe ophthalmoplegia	double CMAP<br>delayed pupillary responses	stridor<br>tongue wasting	associated myopathy (± tubular aggregates)	contractures<br>episodic respiratory crisis<br>strabismus	double CMAP
Treatment response	py -ve<br>3,4-DAP +ve<br>β2AR +ve	py +ve<br>3,4-DAP +ve<br>β2AR +ve	py -ve<br>β2AR +ve	Py -ve<br>β2AR +ve	py +ve<br>3,4-DAP +ve<br>β2AR +ve	py +ve<br>3,4-DAP +ve	fluoxetine or quinidine
Course of disease over time	improves (scoliosis/chest may progress)	stable	slowly progressive	slowly progressive	slowly progressive	improves	slowly progressive

The details provided in this table summarise the most common clinical features but are not meant to be a precise description covering the whole clinical spectrum for each CMS subtype. Clinical features of patients can be highly variable and therefore not be reflected in this table. Py, pyridostigmine; 3,4-DAP, 3,4-Diaminopyridine; β2AR, Beta-2 adrenergic agonists; +ve, positive; -ve, negative.
